# Sterilize Weak-Minded Couples, or Prohibit Marriage, Says Governor of Kentucky

**Published:** 1911-02

**Authors:** 


### Sterilize Weak-Minded Couples, or Prohibit Marriage, Says Governor of Kentucky.

Couples whom nature has not endowed with strong minds or whose offspring would, as a result of such a marriage, be weak-minded, should not be permitted by law to bear offspring, is the opinion of Governor Willson.

"They should be prohibited by law from marriage or subjected to a surgical operation which would prevent offspring," are his words.

Many Governors have held to this opinion privately, but few before Governor Willson have been progressive and fearless enough to announce it. That such a law, if recommended to the Legislature, would be regarded radical there is no doubt, and if there is an extra session of the Legislature in the State such a law may be recommended by Governor Willson, not only to apply to weak-minded people, but for insane and degenerates as well.

Governor Willson came to this conclusion six months ago when reading the record in the case of the Commonwealth against Floyd Frazier, who was hanged for the murder of Mrs. Ellen Flannery at Whitesburg. He hesitated to express it then, but when the case of the Commonwealth against Jim White, the negro boy who is to be hanged at Pineville, January 30th, came before him he decided to keep his views no longer from the public and hopes they will become deep-seated in the minds of thinking people and bear good fruit.

He said: "White is one of the victims of the mating of feeble-minded people, but he is none the less dangerous to society, and his case, if not punished by death, is dangerous to the whole State. In his appeal for clemency, I do not care to consider the question chiefly dwelt on as to the extent of the precise mental caliber of the criminal.

"I believed in the death sentence of Guiteau and Czolgoz, whether mentally wholly sound or not, and I believe death in this case a sacred duty to society as long as our law provides for capital punishment, and this is one of the last classes of cases in which I would abolish it."

Nothing has occurred during Governor Willson's administration that has affected him more than the receipt of a letter from the simple-minded negro White, asking that his sentence be commuted. It follows:

"Mr. Governor Willson—Dear Sir: Will you please release me from the death sentence. This is from James White. All my hope and faith, it is in you and the Lord. Through and by reason of the Lord I have to look to you for all of my earthly mercy.

"I pray to God, day by day, night by night, that you will be merciful upon this condemned body of mine.

"Answer soon, for the sake of the poor little boy.

"JAMES WHITE."

